# Data on sentiments and emotions of olympic-themed tweets

**DOI:** 10.1016/j.dib.2019.103869

**Published:** 2019-03-20

**Authors:** Joshua J. Vertalka, Eva Kassens-Noor, Mark Wilson

**Affiliations:** a Resilient Solutions 21 (RS21), USA; b Michigan State University, USA

**Keywords:** Olympic, R rdata, Twitter, Emotion lexicon

## Abstract

Two code files and one dataset related to Olympic Twitter activity are the foundation for this article. Through Twitter's Spritzer streaming API (Application Programming Interface), we collected over 430 million tweets from May 12th, 2016 to September 12th, 2016 windowing the Rio de Janeiro Olympics and Paralympics. We cleaned and filtered these tweets to contain Olympic-related content. We then analyzed the raw data of 21,218,652 tweets including location data, language, and tweet content to distill the sentiment and emotions of Twitter users pertaining to the Olympic Games Kassens-Noor E. et al., 2019. We generalized the original data set to comply with the Twitter's Terms of Service and Developer agreement, 2018. We present the modified dataset and accompanying code files in this article to suggest using both for further analysis on sentiment and emotions related to the Rio de Janeiro Olympics and for comparative research on imagery and perceptions of other Olympic Games.

**Table:** Specifications table

Subject area	*Social Sciences*
More specific subject area	*Social Media*
Type of data	*Figure*
How data was acquired	*Twitter's Development API, Stream (Spritzer)*
Data format	*R Rdata format*
Experimental factors	*Ran Arguments and Execute code files on raw data*
Experimental features	*Key word analysis; Mohammad and Turney's*[3]*sentiment and emotion lexicon*
Data source location	*East Lansing, USA, Michigan State University*
Data accessibility	*Public repository on Mendeley Data:*https://doi.org/10.17632/w2329m379w.1 *Direct link:*https://data.mendeley.com/datasets/w2329m379w/1
Related research article	Kassens-Noor E., Vertalka J, Wilson M. (2019). “Good Games, bad host? Using big data to measure public attention and imagery of the Olympic Games” *Cities.* 90, pp. 229–236 https://doi.org/10.1016/j.cities.2019.02.009[1]

**Table:** 

**Value of the data** • Code files can be easily adopted to extract similar raw data sets from life streaming Twitter APIs • Data can be used to measure the magnitude and sentiments of Olympic related Twitter activity before during and after the Rio de Janeiro Olympic Games • Code files can be used for scoring of sentiments and emotions of Olympic themed tweets • Data can be used for comparisons related to Olympic Games' imagery between Twitter and other Social Media

## 1 Data

**Fig. 1:**
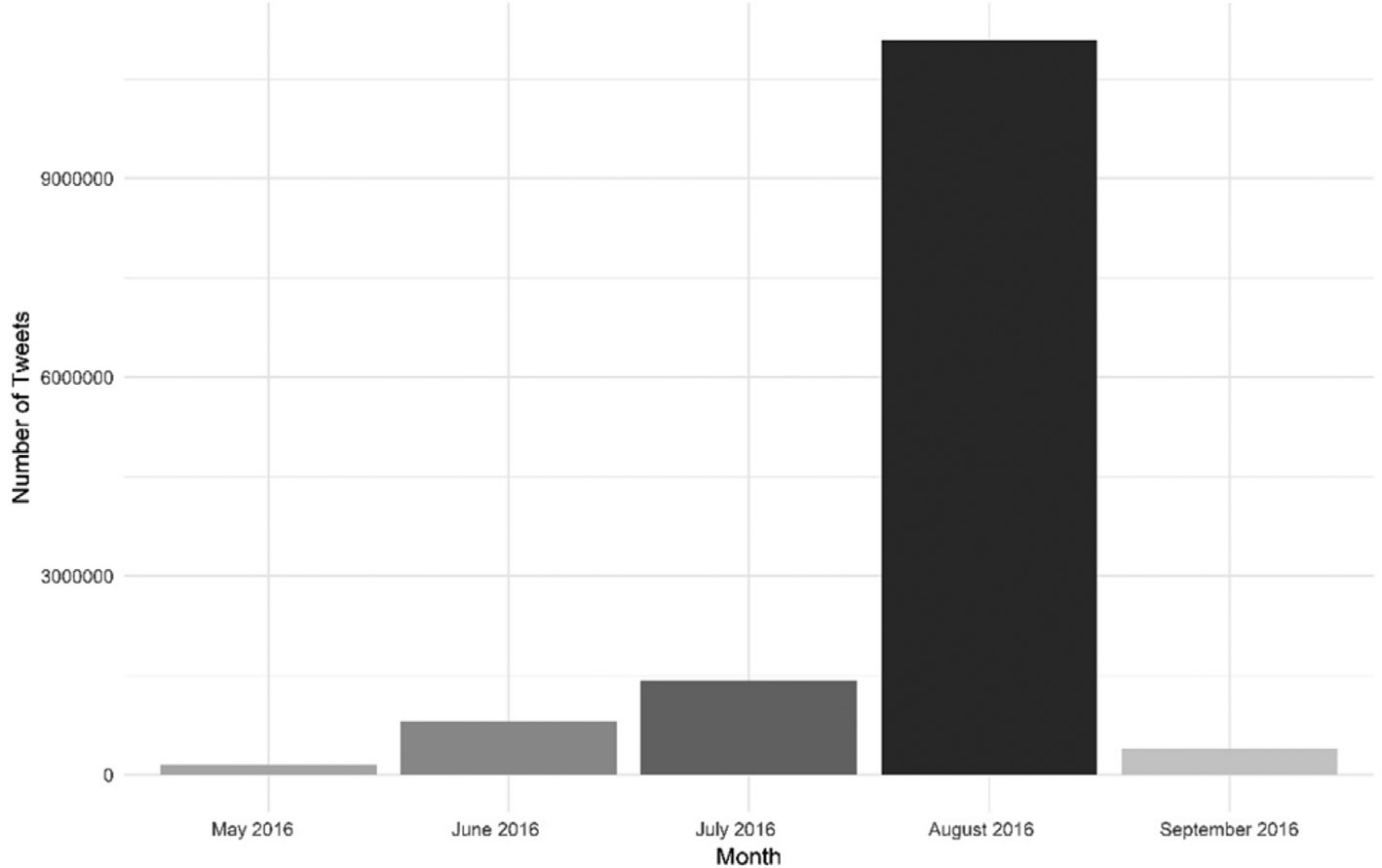
Number of Tweets by Month in 2016. Note: Rio de Janeiro Olympic Games (5-21 August 2016).

The Rdata file contains 13,849,198 coded tweets distilling the magnitude, emotion, and sentiments related to the Olympic Games. For example, Fig. 1 shows the monthly distribution of the 13,849,198 tweets associated with Olympic hashtags between May 12th, 2016 and September 12th, 2016. Our dataset can be further used to show the magnitude of attention and scope of sentiments and emotions related to the Olympic Games and their host cities before, during, and after the Rio de Janeiro Olympic Games. The data has been filtered to remove any raw data including tweets, retweets, and Twitter handles to comply with the Twitter Terms of Service licensing agreement and copyright [2]. The Mendeley dataset (Databrief – Data) contains the following columns: assigned number of tweet, time of tweet, date of tweet, verification of Olympic-related hashtag search, longitude of post (if applicable), latitude of post (if applicable), as well as anger, anticipation, disgust, fear, joy, sadness, surprise, anger, trust, positive sentiment, and negative sentiment codes for each English tweet.

The Olympic Games increase visibility of host cities and broadcast the host's imagery across the world [1], [4], [5]. The use of social media and especially Twitter to analyze public discourse related to the Olympics is a rapidly evolving topic [6], [7], while associated datasets [8] are rare yet of extreme importance to discern changes in the public discourse about mega-events. This data article fills this important data need, while our related article describes sentiment and emotions for the Olympic Games and their host cities [1].

### 1.1 Experimental design, materials, and methods

Tweets were downloaded from Twitter's Application Program Interface (Spritzer Stream) from May 12th, 2016 to September 12th, 2016 based on Olympic Game keywords and associated hashtags. We searched for hashtags related to all modern Summer and Winter Olympic Games, but only the following captured tweets: Olympic (Olympics/#olympic/#olympics/Olympic Games/Olympic), Rio (Rio2016/#Rio2016), Tokyo (Tokyo2020/#Tokyo2020), London (London2012/#London2012), International Olympic Committee (IOC/#IOC), Los Angeles (LA2024/#LA2024), Beijing (Beijing2008/#Beijing2008/Beijing2022/#Beijing2022), Paris (Paris2024/#Paris2024), Sydney (Sydney2000/#Sydney2000), Budapest (Budapest2024/#Budapest2024), Atlanta (Atlanta1996/#Atlanta1996), Athens (Athens2004/#Athens2004), Rome (Rome2024/#Rome2024), Sochi (Sochi2014/#Sochi2014), Barcelona (Barcelona1992/#Barcelona1992), Seoul (Seoul1988/#Seoul1988), Vancouver (Vancouver2010/#Vancouver2010), Hamburg (Hamburg2024/#Hamburg2024), Turin (Turin2006/#Turin2006), Calgary (Calgary1988/#Calgary1988), Salt Lake City (SaltLakeCity2002/#SaltLakeCity2002), and Pyeongchang (Pyeongchang2018/#Pyeongchang2018).

Code file Execute and associated Code file Argument filtered the captured tweets to (a) remove retweets and duplicate tweets (b) search for and record Olympic Game keywords for all remaining tweets (c) search for and record all Sentiment and emotion keywords for every tweet based on Mohammand and Turney's [3] sentiment and emotion lexicon. Code files Execute and Argument can be modified to analyze other events and can be applied jointly with Twitter's APIs to extract a similar raw data set from live-streaming Twitter feeds.
